# LRG1 and SDR16C5 protein expressions differ according to HPV status in oropharyngeal squamous cell carcinoma

**DOI:** 10.1038/s41598-024-64823-w

**Published:** 2024-06-19

**Authors:** Reija Randén-Brady, Timo Carpén, Laura C. Hautala, Tuomas Tolvanen, Caj Haglund, Sakari Joenväärä, Petri Mattila, Antti Mäkitie, Sanna Lehtonen, Jaana Hagström, Suvi Silén

**Affiliations:** 1 Department of Pathology, University of Helsinki, 00014 Helsinki, Finland; 2 Department of Otorhinolaryngology—Head and Neck Surgery, University of Helsinki, and Helsinki University Hospital, 00029 Helsinki, Finland; 3 Faculty of Medicine, Research Program in Systems Oncology, University of Helsinki, 00014 Helsinki, Finland; 4 Research Program for Clinical and Molecular Metabolism, University of Helsinki, 00014 Helsinki, Finland; 5 University of Helsinki, and Helsinki University Hospital, 00029 Helsinki, Finland; 6 Research Programs Unit, Translational Cancer Medicine, University of Helsinki, 00014 Helsinki, Finland; 7 Division of Ear, Nose and Throat Diseases, Department of Clinical Sciences, Intervention and Technology, Karolinska Institute and Karolinska Hospital, 171 76 Stockholm, Sweden; 8 Department of Oral Pathology and Oral Radiology, University of Turku, 20520 Turku, Finland; 9 Transplantation Laboratory, Haartman Institute, University of Helsinki and Helsinki University Hospital, 00029 Helsinki, Finland; 10 HUS Diagnostic Center, Department of Pathology, HUSLAB, Helsinki University Hospital, 00029 Helsinki, Finland; 11 Department of Pathology, University of Helsinki and Helsinki University Hospital, 00290 Helsinki, Finland

**Keywords:** Head and neck cancer, Diagnostic markers, Predictive markers, Prognostic markers, Cancer, Cancer, Biomarkers, Diseases

## Abstract

The increasing incidence of oropharyngeal squamous cell carcinoma (OPSCC) is primarily due to human papillomavirus, and understanding the tumor biology caused by the virus is crucial. Our goal was to investigate the proteins present in the serum of patients with OPSCC, which were not previously studied in OPSCC tissue. We examined the difference in expression of these proteins between HPV-positive and -negative tumors and their correlation with clinicopathological parameters and patient survival. The study included 157 formalin-fixed, paraffin-embedded tissue samples and clinicopathological data. Based on the protein levels in the sera of OPSCC patients, we selected 12 proteins and studied their expression in HPV-negative and HPV-positive OPSCC cell lines. LRG1, SDR16C5, PIP4K2C and MVD proteins were selected for immunohistochemical analysis in HPV-positive and -negative OPSCC tissue samples. These protein´s expression levels were compared with clinicopathological parameters and patient survival to investigate their clinical relevance. LRG1 expression was strong in HPV-negative whereas SDR16C5 expression was strong in HPV-positive tumors. Correlation was observed between LRG1, SDR16C5, and PIP4K2C expression and patient survival. High expression of PIP4K2C was found to be an independent prognostic factor for overall survival and expression correlated with HPV-positive tumor status. The data suggest the possible role of LRG1, SDR16C5 and PIP4K2C in OPSCC biology.

## Introduction

Like other head and neck squamous cell carcinomas, oropharyngeal squamous cell carcinoma (OPSCC) has traditionally been linked to alcohol and tobacco consumption^1,2^. In recent decades, however, the number of OPSCC cases associated with human papillomavirus (HPV) has increased significantly and is often seen in healthier, younger, and non-smoking patients^3,4^, and HPV is now recognized as one of the most significant risk factors for OPSCC – up to 90% of OPSCC cases are linked to HPV infection^5,6^. At the molecular level, many differences have been demonstrated to be associated with HPV status in OPSCC patients^7–10^. The most studied protein in oropharyngeal tumors is p16, a surrogate marker for HPV, and its related molecular pathways. Finding other proteins whose expression alters in relation to HPV status could help to understand changes in tumor biology caused by the virus^11^.

We aimed at identifying new proteins expressed in OPSCC tissue and finding out whether their expression level is different in HPV-positive and -negative tumors. In our previous study, we compared the serum protein levels of HPV-positive and -negative OPSCC patients^12^. From this comparison, we selected proteins to now be studied on tissue samples. We first studied their expression in OPSCC cell lines to confirm their presence in tumor cells. HaCat cell line (immortalized keratinocytes) was used as a benign epithelial cell control. We selected four of the 12 proteins for further immunohistochemical (IHC) studies in patient samples based on protein expression in cell lines to assess their potential clinical relevance. The four proteins selected for closer examination were Leucine-rich-α-2-glycoprotein 1 (LRG1), Short chain dehydrogenase/reductase family 16C member 5 (SDR16C5), Phosphatidylinositol-5-phosphate 4-kinase type 2 lipid kinase (PIP4K2C) and Mevalonate diphosphate decarboxylase (MVD).

LRG1 is a secreted glykoprotein, a member of the Leucine-rich repeat (LRR) family of proteins, which has been involved in many essential biological processes^13^. It has been reported to play a role in inflammatory responses such as in autoimmune disorder rheumatoid arthritis^14^ and ulcerative colitis^15^. It also has been discovered that LRG1 is involved in the regulatory mechanism of abnormal angiogenesis by adjusting endothelial TGF-β signaling^16^. Unbalanced neovascularization contributes to tumor growth and LRG1 has been reported to be upregulated in many cancer types^17–20^. Furthermore, LRG1 has been studied in head and neck cancer, but previous studies did not include OPSCC patients^21^.

SDR16C5 is a member of the short-chain alcohol dehydrogenases/reductases protein family, catalysing the first and rate-limiting step that produces retinaldehyde from retinol via oxidation^22,23^. SDR16C5 is an integrated intracellular protein locating on the endoplasmic reticulum in mature cells^24^. SDR16C5 is suggested to play a role in the oxidation of retinol to retinaldehyde and retinoic acid biosynthesis in keratinocytes^24^.

PIP4K2C is an intracellular protein located endoplasmic reticulum and cytoplasm being member of lipid kinase family. Proteins of PIP4K2 family have been shown to play an important role in the regulation of oxidative stress signalling and normal cell development^25^.

MVD is an intracellular protein participating in the mevalonate pathway of cholesterol biosynthesis, which is part of polyisoprenoid and sterol biosynthesis^26^. The isoprenylated proteins regulate cell growth, division, and differentiation, and are more likely to be associated with the retained nuclei in the stratum corneum, like in parakeratosis^27^. The regulation of the mevalonate metabolic pathway is demonstrated by the fact that high activity can result in malignant transformation while low activity inhibits cellular function and survival^28^.

In our study we compared a set of proteins selected based on their expression in the serum of HPV-negative and HPV-positive OPSCC patients. First, we compared protein expression in a benign control cell line (HaCat) and in HPV-positive and HPV-negative cell lines. From this comparison, we selected the proteins whose expression we examined by IHC in the tissue samples. The four proteins selected for closer examination were LRG1, SDR16C5, PIP4K2C and MDV. All these proteins have also been previously studied in malignancies, but to our knowledge not yet in OPSCC.

## Materials and methods

### Patient material

The series contained tissue samples from 157 OPSCC patients diagnosed with OPSCC during a 4-year period between 2012 and 2016 at the Department of Otorhinolaryngology – Head and Neck Surgery, Helsinki University Hospital, Helsinki, Finland. Clinicopathological data including sex, age at diagnosis, smoking status, alcohol usage, tumor’s T class, N class, stage, and grade were gathered from hospital records. To determine tumor stage and TNM class, the 8th edition of the American Joint Committee on Cancer (AJCC) Staging Manual^29^ was used. The sample materials are partly the same as those reported in our previous studies^12,30^.

### Protein selection

**Figure 1:**
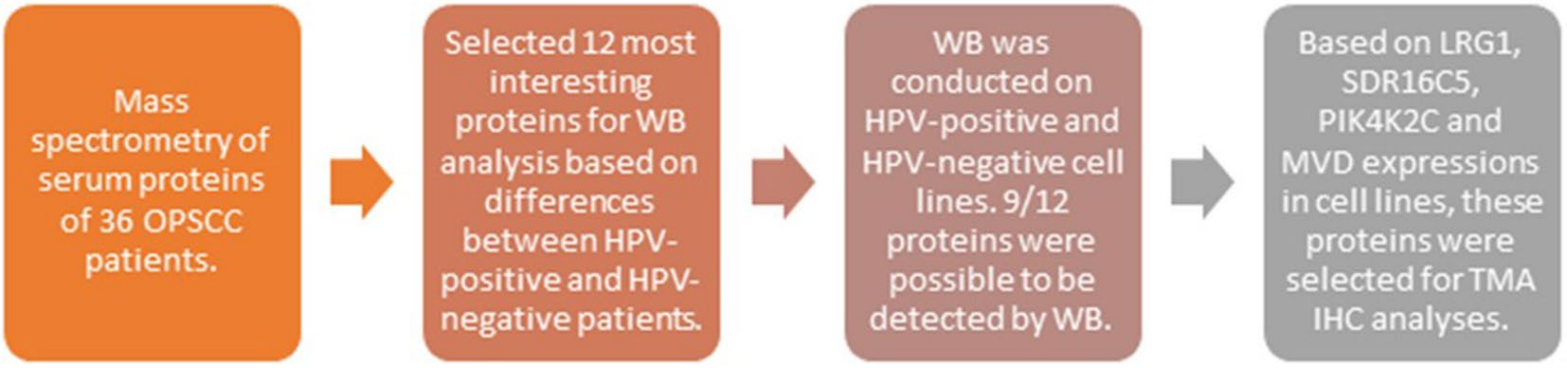
Workflow of material selection. Abbreviations: *OPSCC* oropharyngeal squamous cell carcinoma, *HPV* human papillomavirus, *TMA* tissue microarray, *WB* western blot, *IHC* immunohistochemistry.

Based on our previous study^12^ we selected proteins for further analyses in a patient series with tissue material available. Selection of proteins for further analysis is described in (Fig. 1).

### Tissue material

Tissue microarray (TMA) blocks were prepared from formalin-fixed paraffin-embedded FFPE samples using a methodology described in our earlier studies^30,31^. To produce TMA blocks of the hematoxylin- eosin (HE)-stained tumor tissue samples, the tumor areas were annotated. Six representative core punctuates (one mm in diameter) of each tumor were detached and placed into a paraffin block with a tissue microarrayer (Beecher Instruments, Silver Spring, MD, USA). Tumor tissue material contained 157 FFPE samples on TMA blocks. The patients who had positive results in tissue samples for both immunohistochemical detection of p16^INK4a^ and in situ hybridization for high-risk HPV E6/E7 mRNA (HPV RNA ISH) were counted as HPV positive. p16 immunohistochemistry and HPV RNA ISH methods have previously been described^30,31^.

### Cell lines

Immortalized human keratinocyte benign cell lines (HaCat) were grown in Dulbecco's Modified Eagle Medium (DMEM) (Sigma-Aldrich, St. Louis, MO, USA) supplemented with 5% FBS (Sigma-Aldrich), glutamine (Lonza, Walkersville, MD, USA), penicillin–streptomycin (Sigma-Aldrich), and non-essential amino acids (NEAA) (Gibco / Thermo Fisher Scientific, Waltham, Massachusetts, USA). Two human OPSCC lines (SCC-65 HPV negative, p16 negative and SCC-69 HPV positive, p16 positive) from the Department of Otorhinolaryngology—Head and Neck Surgery, University of Turku, were grown in high glucose DMEM (Sigma-Aldrich) supplemented with 10% FBS, glutamine, penicillin–streptomycin, NEAA, and sodium pyruvate (Gibco / Thermo Fisher Scientific). All the cell lines were cultured at + 37 °C in a humidified incubator with 5% CO_2_.

### Western blotting

For Western blotting, HaCat SCC-65 and SCC-69 cells were lysed using NP-40 buffer (1% nonidet P-40, 20 mM Tris–HCl pH 7.4, 150 mM NaCl) supplemented with a protease inhibitor cocktail (cOmplete Protease Inhibitor Cocktail tablets, Roche Diagnostics GmbH, Mannheim, Germany) and phosphatase inhibitors (50 mM NaF and 1 mM Na_3_VO_4_) after which 30 µg of each cell lysate was separated in 8% polyacrylamide gels and transferred onto Immobilon-FL PVDF membrane (Merck Millipore Ltd., Tullagreen, Ireland). In order to probe for other proteins from the same membrane, the membranes were cut into smaller strips before incubating with the primary antibodies. The membranes were blocked using Intercept® (TBS) Blocking Buffer (LI-COR, Lincoln, NE, USA), incubated with rabbit anti-LRG1 (HPA001888)^16^, anti-PIP4K2C (HPA028658)^32^, anti-SDR16C5 (HPA025224)^33^, anti-MVD (HPA048250)^34^ IgGs (Atlas Antibodies, Stockholm, Sweden) and mouse anti-β-actin (A3853) IgGs (Sigma-Aldrich) followed by secondary antibodies (IRDye 800CW Donkey anti-rabbit/anti-mouse and IRDye 680RD Donkey anti-mouse IgGs, all from LI-COR and Alexa Fluor 680 Donkey anti-rabbit IgG from Invitrogen, Carlsbad, CE, USA). The membranes were scanned using Odyssey Infrared Imaging System (LI-COR) and quantified with Image Studio software (LI-COR).

### Immunohistochemical detection of SDR16C5, PIP4K2C, LRG1 and MVD

TMA blocks were cut into four-micrometre-thick tissue slides and baked at + 56 °C for one hour. Deparaffinization, rehydration, and antigen retrieval steps were performed in one step with a 3-in-1 specimen preparation procedure. EnVision FLEX Target Retrieval Solution, high pH 9.0 (Agilent Dako, CA, United States) was used for SDR16C5 (HPA025224, Atlas Antibodies, Sweden), PIP4K2C (HPA028658, Atlas Antibodies, Sweden) and LRG1 (HPA001888, Atlas Antibodies, Sweden) antibodies in DAKO PT Link for pre-treatment (Agilent Dako, CA, United States). For MVD (HPA048250, Atlas Antibodies, Sweden), antibody EnVision FLEX Target Retrieval Solution, low pH 6.0 (Agilent Dako, CA, United States) was used. Stainings were performed in an Autostainer 480 instrument (LabVision Corp., Fremont, CA, United States). The immunoreaction detection system was DAKO Envision Flex Detection System kit (Agilent Dako, CA, United States) and the signal was enhanced with Envision Flex Mouse/Rat linker (Agilent Dako, CA, United States) at PIP4K2C and LRG1 stainings. To reduce background staining, DAKO Real Peroxidase Blocking Solution (Agilent Dako, CA, United States) was applied. Primary antibodies were incubated overnight at + 4 °C with the following dilutions: SDR16C5, LRG1 and MVD to 1:100 and PIP4K2C to 1:300. The visualization was performed with DAB and slides were counterstained with Envision Flex Heamatoxyline (Agilent Dako, CA, United States). Finally, slides were dehydrated, mounted, and cover slipped.

### Scoring of immunohistochemical stainings

Immunopositivity was analysed by applying a scoring method modified from previous studies^35^, where valuation of the markers included intensity of staining and frequency of positive tumor cells. Intensity of staining was scored as (0) no staining, (1) weak, (2) moderate, and (3) strong. Based on the percentage of positive tumor cells, frequency was evaluated as (0) < 5%, (1) 5–10%, (2) 11–50%, and (3) > 50%. Two scoring results were multiplied for each sample, resulting in a composite immunoreactive score IRS = intensity x frequency that had a range between 0–9. Expression of antibodies was categorized as (0) negative, (1–3) weak, (4–6) moderate, and (7–9) strong positive.

### Statistical analysis

All Western blotting data were presented as mean ± SEM of three individual experiments (n = 9 for each cell line). For statistical analyses, unpaired, two-tailed t-test was performed with a limit of significance set to 0.05 using GraphPad Prism 8 (GraphPad Software, Inc., San Diego, CA, USA).

Statistical data analyses for immunohistochemical staining were performed using IBM SPSS Statistics version 27 (IBM Corp.) Overall, *p*-values < 0.05 were regarded as statistically significant. One-Way ANOVA was used to compare age of continuous variables and IHC scoring results. To analyze clinicopathological variables and scoring results, crosstabulation Chi-square test and Independent-Samples Mann–Whitney U Test and Independent samples t-test was used. Kaplan–Meier survival analyses and log-rank test (Mantel-Cox) was used to estimate the 5-year disease-specific survival (DSS) and overall survival (OS). DSS was defined as time from end of treatment to death caused by target OPSCC. OS was defined as time from end of treatment to death from any cause. For the survival statistics, 15 patients with palliative treatment intent were excluded. Cox regression analyzes were used for measuring independent prognostic value of the immunohistochemical markers.

### Ethics approval

Study was approved by the the institutional Research Ethics Board at the Helsinki University Hospital (DNr. 51/13/03/02/2013) and a study permission was granted. Informed written consent was requested from all patients and the study followed the Declaration of Helsinki 2013.

## Results

### Protein expression on OPSCC and benign cell lines

**Figure 2:**
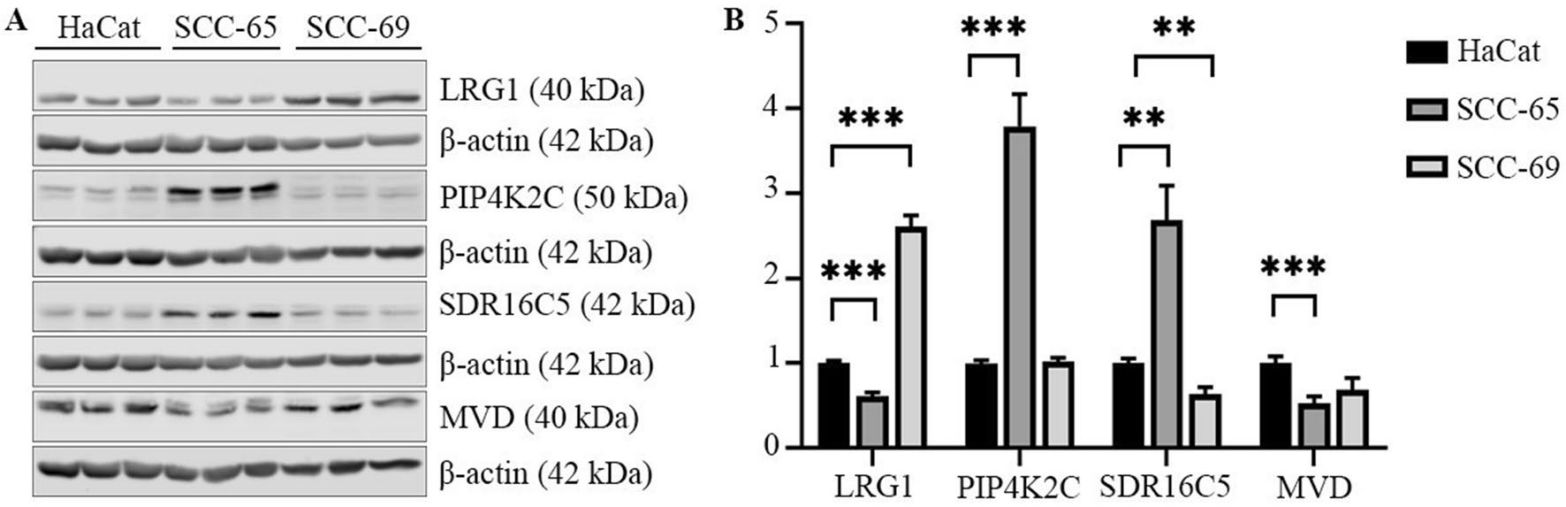
Expression analysis of the four selected proteins LRG1, PIP4K2C, SDR16C5 and MVD in control, HPV-negative and HPV-positive cell lines. (**A**) Representative Western blots of LRG1, PIP4K2C, SDR16C5 and MVD in lysates of HaCat, SCC-65 and SCC-69 cells. Original Western blot membranes are available in Supplementary Fig. 1. (**B**) The expression levels of LRG1, PIP4K2C, SDR16C5 and MVD quantified from immunoblots as in (**A**) and normalized to actin. Benign HaCat, Human immortalized keratinocyte cell line; Human OPSCC SCC-65, HPV-negative cell line; SCC-69, HPV-positive cell line. Data are presented as mean ± SEM; ** < 0.01, ***p < 0.001.

LRG1 was strongly expressed in the HPV-positive cell line (SCC-69, p = 7.82 × 10^–7^) whereas SRD16C5 and PIP4K2C had stronger expression in the HPV-negative cell line (SCC-65, p = 0.003 and p = 7.61 × 10^–5^, respectively) compared to a benign keratinocyte cell line (HaCat). HaCat cell line (immortalized keratinocytes) is at the moment widely used and the only benign epithelial cell line available for control. MVD was strongly expressed in HaCat-cell line compared to SCC-cell lines. Expression results are shown in (Fig. 2, Supplementary Fig. 1). In addition, we studied Centromere protein S (APITD1), Annexin A1 (ANXA1), Galectin 3 binding protein (LGALS3BP), Serpin family G member 1 (SERPING)1 and FERM, ARH/RhoGEF and pleckstrin domain protein 2 (FRAP2) proteins with Western blotting. Their expression levels did not significantly differ in HPV-positive and HPV-negative cell lines and were excluded from further analyses.

### Protein localization on OPSCC tissue samples

**Figure 3:**
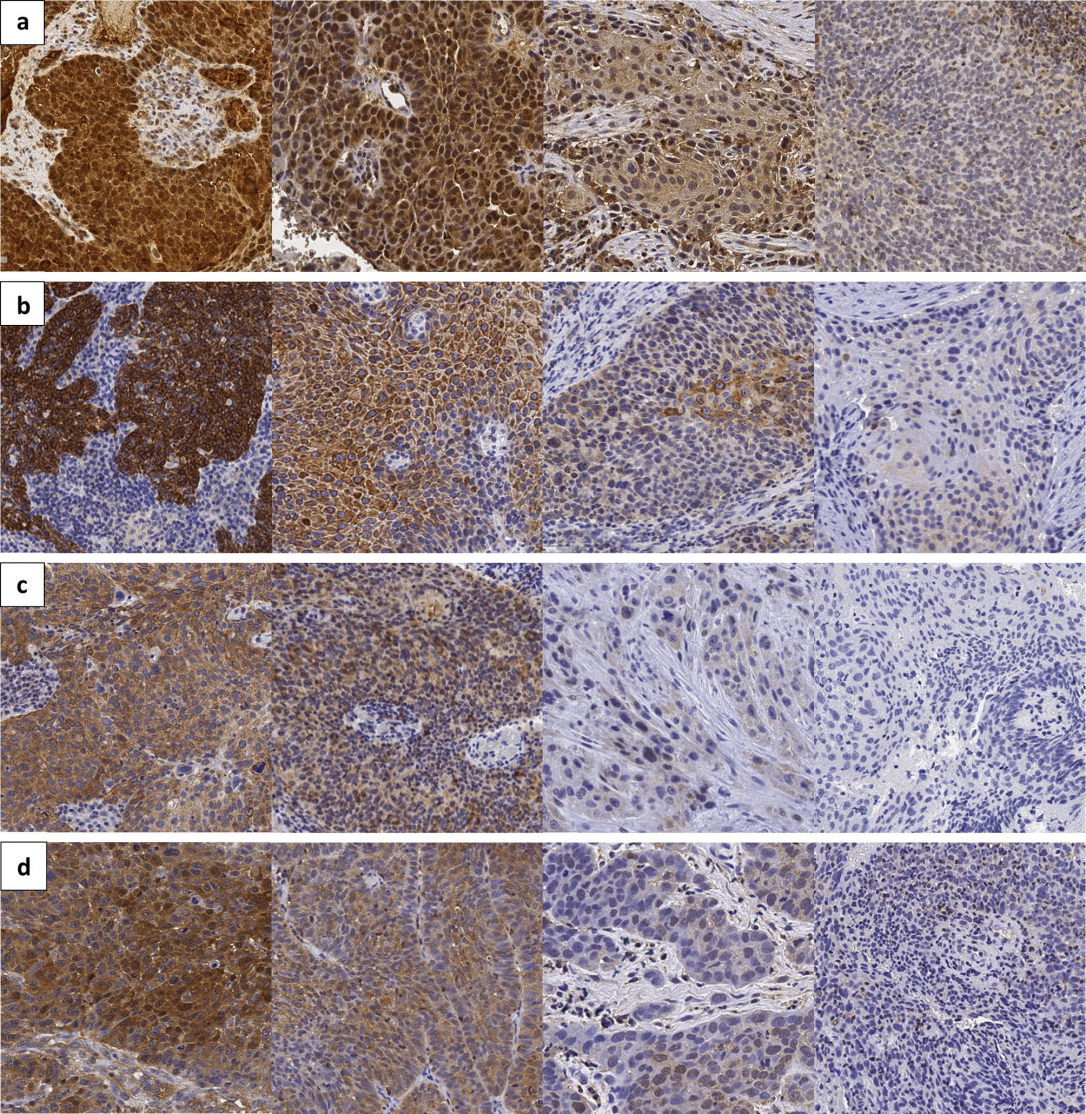
Protein expressions in OPSCC tissue samples by immunohistochemistry. Strong, moderate, weak, and negative expression in studied biomarkers (**a**) LRG1 (**b**) SDR16C5 (**c**) PIP4K2C and d) MVD expression. Scale bar 100 µm, original magnification 40x.

As LRG1, SDR16C5, PIP4K2C and MVD expression levels clearly differed between HPV-positive and HPV-negative OPSCC cell lines, they were selected for an immunohistochemistry study utilizing tissue samples of 157 OPSCC patients. Although there were 157 samples in total, not every patient had a representative sample retained in TMA sections for every staining, causing variation in sample results for immunohistochemical markers. All proteins localized in the cytoplasm (Fig. 3).

### Association of immunohistochemical markers and HPV status

We compared the expression levels of LRG1, SDR16C5, PIP4K2C and MVD in HPV-positive and HPV-negative tumors (Table 1). Of the 157 patient samples, 103 (66%) were HPV positive and 54 (34%) HPV negative. The expression levels of PIP4K2C and MVD did not correlate with the HPV status of the tumor (Table 1). Notably, in HPV-negative tumors, strong LRG1 expression was detected in 17.6% of cases while in HPV-positive tumors, strong LRG1 expression was observed only in 5% of cases (*p* = *0.001*). Also, SDR16C5 expression differed between the HPV status: 26% of the HPV-positive tumors strongly expressed SDR16C5, while strong expression was observed in only 11.1% of HPV-negative tumors (*p* = *0.015*).

**Table 1:** Expression of immunohistochemical markers according to HPV status.

Variable	HPV pos	%	HPV neg	%	Total	p-value
LRG1						
Negative	9	9.9	0	0.0		
Weak	38	38.0	7	13.7		
Moderate	48	48.0	35	68.6		
Strong	5	5.0	9	17.6		
Total	100		51		151	** < *0.001***
SDR16C5						
Negative	5	5.0	9	16.7		
Weak	22	22.0	12	22.0		
Moderate	47	47.0	27	50.0		
Strong	26	26.0	6	11.1		
Total	100		54		154	***0.015***
PIP4K2C						
Negative	3	2.9	2	3.8		
Weak	27	26.2	19	36.5		
Moderate	56	54.4	21	40.4		
Strong	17	16.5	10	19.2		
Total	103		52		155	***0.413***
MVD						
Negative	36	35.6	12	23.5		
Weak	29	28.7	23	45.1		
Moderate	30	29.7	12	23.5		
Strong	6	5.9	4	7.8		
Total	101		51		152	***0.528***

Significant values are in bold.

### Associations of immunohistochemical markers with clinicopathological characteristics

We compared LRG1, SDR16C5, PIP4K2C and MVD expression levels to specific clinicopathological characteristics. Patient characteristics and histopathological findings are described in Table 2 and Table 3.

**Table 2:** Clinicopathological parameters and associations to immunohistochemical markers.

Variable	LRG1 (%)				p-value	SDR16C5 (%)				p-value	PIP4K2C (%)				p-value	MVD (%)				p-value
Score	0	1	2	3		0	1	2	3		0	1	2	3		0	1	2	3	
Number of patients	9	45	83	14	151	14	34	74	32	154	5	46	77	27	155	48	52	42	10	152
Mean age at diagnosis	59.3	61.1	62.6	61.9	***0.663***	62.2	62.6	62.5	61.0	***0.764***	62.0	63.1	61.9	60.1	0.596	62.3	61.4	61.7	62.5	***0.947***
Sex					***0.636***					***0.850***					***0.991***					***0.770***
Male	7 (6.2)	36 (31.9)	58 (51.3)	12 (10.6)	113	8 (6.9)	28 (24.1)	58 (50.0)	22 (19.0)	116	4 (3.4)	33 (28.4)	60 (51.7)	19 (16.4)	116	37 (32.2)	39 (33.9)	32 (27.8)	7 (6.1)	115
Female	2 (5.3)	9 (23.7)	25 (65.8)	2 (5.3)	38	6 (15.8)	6 (15.8)	16 (42.1)	10 (26.3)	38	1 (2.6)	13 (33.3)	17 (43.6)	8 (20.5)	39	11 (29.7)	13 (35.1)	10 (27.0)	3 (8.1)	37
Total	9 (6.0)	45 (29.8)	83 (55.0)	14 (9.3)	151	14 (9.1)	34 (22.1)	74 (48.1)	32 (20.8)	154	5 (3.2)	46 (29.7)	77 (49.7)	27 (17.4	155	48 (31.6)	52 (34.2)	42 (27.6)	10 (6.6)	152
Smoking					***0.002***					***0.276***					***0.852***					***0.537***
Non-smoker	5 (13.2)	15 (39.5)	18 (47.4)	0 (0.0)	38	2 (5.4)	8 (21.6)	18 (48.6)	9 (24.3)	37	2 (5.1)	11 (28.2)	19 (24.7)	7 (17.9)	39	19 (50.0)	4 (10.5)	11 (28.9)	4 (10.5)	38
Ex-smoker	2 (3.9)	18 (35.3)	26 (51.0)	5 (9.8)	51	6 (11.1)	8 (14.8)	27 (50.0)	13 (24.1)	54	2 (3.8)	17 (32.1)	25 (47.2)	9 (17.9)	53	15 (28.8)	21 (40.4)	13 (25.0)	3 (5.8)	52
Current smoker	2 (3.2)	12 (19.4)	39 (62.9)	9 (14.5)	62	6 (9.5)	18 (28.6)	29 (46.0)	10 (15.9)	3	1 (1.6)	18 (48.7)	33 (52.4)	11 (17.5)	63	14 (22.6)	27 (43.5)	18 (29.0)	3 (4.8)	62
Total	9 (6.0)	45 (29.8)	83 (55.0)	14 (9.3)	151	14 (9.1)	34 (22.1)	74 (48.1)	32 (20.8)	154	5 (3.2)	46 (29.7)	77 (49.7)	27 (17.4)	155	48 (31.6)	52 (34.2)	42 (27.6)	10 (6.6)	152
Alcohol use					***0.039***					***0.725***					***0.851***					***0.574***
Never	4 (5.6)	23 (31.9)	41 (56.9)	4 (5.6)	*72*	7 (9.9)	12 (16.9)	34 (47.9)	18 (25.4)	*71*	4 (5.6)	17 (23.6)	40 (55.6)	11 (15.3)	*72*	23 (31.9)	24 (33.3	18 (25.0)	7 (9.7)	72
Earlier	0 (0.0)	3 (15.0)	13 (65.0)	4 (20.0)	20	4 (19.9)	5 (23.8)	9 (42.9)	3 (14.3)	21	0 (0.0)	7 (33.3)	10 (47.6)	4 (19.0)	21	6 (28.6)	11 (52.4)	4 (19.0)	0 (0.0)	21
Currently	1 (2.9)	9 (25.7)	19 (54.3)	6 (17.1)	35	4 (10.8)	9 (24.3)	18 (48.6)	6 (16.2)	37	2 (5.4)	11 (29.7)	17 (45.9)	7 (18.9)	37	11 (32.4)	10 (29.4)	10 (29.4)	3 (8.8)	34
Total	5 (3.9)	35 (27.6)	73 (57.5)	14 (11.0)	127	15 (11.6)	26 (20.2))	61 (47.3)	27 (20.9)	129	6 (4.6)	35 (26.9)	67 (51.5)	22 (16.9)	130	40 (31.5)	45 (35.4)	32 (25.2)	10 (7.9)	127

Significant values are in bold.

**Table 3:** Clinicopathological parameters and association to immunohistochemical markers.

Variable	LRG1 (%)				p-value	SDR16C5 (%)				p-value	PIP4K2C (%)				p-value	MVD (%)				p-value
Score	0	1	2	3		0	1	2	3		0	1	2	3		0	1	2	3	
T class					***0.495***					***0.289***					***0.298***					***0.027***
T1-T2	6 (6.6)	30 (33.3)	45 (49.5)	10 (11.0)	91	7 (7.7)	19 (20.9)	44 (48.4)	21 (23.1)	91	3 (3.2)	30 (32.3)	46 (49.5)	14 (15.1)	93	33 (35.5)	35 (37.6)	21 (22.6)	4 (4.3)	93
T3-T4	3 (5.0)	15 (25.9)	38 (63.3)	4 (6.7)	60	7 (11.1)	15 (23.8)	30 (47.6)	11 (17.5)	63	2 (3.2)	16 (25.8)	31 (50.0)	13 (21.0)	62	15 (25.4)	17 (28.8)	21 (35.6)	6 (10.2)	59
Total	9 (6.0)	45 (29.8)	83 (55.0)	14 (9.3)	151	14 (9.1)	34 (22.1)	74 (48.1)	32 (20.8)	154	5 (3.2)	46 (29.7)	77 (49.7)	27 (17.4)	155	48 (31.6)	52 (34.2)	42 (27.6)	10 (6.6)	152
N class					***0.025***					***0.987***					***0.901***					***0.045***
N0	0 (0.0)	4 (15.4)	19 (73.1)	3 (11.5)	26	2 (7.7)	5 (19.2)	15 (57.7)	4 (15.4)	26	0 (0.0)	8 (30.8)	15 (57.7)	3 (11.5)	26	4(15.4)	10 (38.5)	9 (34.6)	3 (11.5)	26
NX	9 (7.2)	41 (32.8)	64 (51.2)	11 (8.8)	125	12(9.4)	29 (22.7)	59 (46.1)	28 )21.9)	128	5 (3.9)	38 (29.5)	62 (48.1)	24 (18.6)	129	44 (34.9)	42 (33.3)	33 (26.2)	7 (5.6)	126
Total	9 (6.0)	45 (29.8)	83 (55.3)	14 (9.3)	151	14 (9.1)	34 (22.2)	74 (48.1)	32 (20.8)	154	5 (3.2)	46 (29.7)	77 (49.7)	27 (17.4)	155	48 (31.6)	52 (34.2)	42 (27.6)	10 (6.6)	152
Stage					***0.033***					***0.064***					***0.343***					***0.106***
I-II	6 (6.3)	35 (36.8)	46 (48.4)	8 (8.4)	95	5 (5.2)	21 (21.9)	47 (49.0)	23 (24.0)	96	2 (2.0)	26 (26.5)	54 (55.1)	16 (16.3)	98	34 (35.1)	34 (35.1)	24 (24.7)	5 (5.2)	97
III-IV	3 (5.4)	10 (17.9)	37 (66.1)	6 (10.7)	56	9 (15.5)	13 (22.4)	27 (46.6)	9 (15.5)	58	3 (5.3	20 (35.1)	23 (40.4)	11 (19.3)	57	14 (25.5)	18 (2.7)	18 (32.7)	5 (9.1)	55
Total	9(6.0)	45 (29.8)	83 (55.0)	14 (9.3)	151	14 (9.1)	34 (22.1)	74 (48.1)	32 (20.8)	154	5 (3.2)	46 (29.7)	77 (49.7)	27 (17.4)	155	48 (31.6)	52 (34.2)	42 (27.6)	10 (6.6)	152
Grade					***0.015***					***0.738***					***0.449***					***0.190***
I	0 (0.0)	0 (0.0)	1 (33.3)	2 (66.7)	3	1 (33.3)	0 (0.0)	2 (66.7)	0 (0.0)	3	0 (0.0)	0 (0.0)	2 (66.7)	1 (33.3)	3	0 (0.0)	2 (66.7)	1 (33.3)	0 (0.0)	3
II	0 (0.0)	6 (20.7)	21 (72.4)	2 (6.9)	29	2 (6.7)	5 (16.7)	20 (66.7)	3 (10.0)	30	1 (3.3)	9 (30.0)	14 (46.7)	6 (20.0)	30	7 (23.3)	8 (26.7)	13 (43.3)	2 (6.7)	30
III	9 (7.6)	39 (32.8)	61 (51.3)	10 (8.4)	119	11 (9.1)	29 (24.0)	52 (43.0)	29 (24.9)	121	4 (3.3)	37 (30.3)	61 (50.0)	20 (16.4)	122	41 (34.5)	42 (35.3)	28 (23.5)	8 (6.7)	119
Total	9 (6.0)	45 (29.8)	83 (55.0)	14 (9.3)	151	14 (9.1)	34 (22.1)	74 (48.1)	32 (20.8)	154	5 (3.2)	46 (29.7)	77 (49.7)	27 (17.4)	155	48 (31.6)	52 (34.2)	42 (27.6)	10 (6.6)	152

Significant values are in bold.

***LRG1***: Immunohistochemical results from 151 samples were available from a total of 157 samples. A correlation was found between LRG1 expression and smoking (*p* = *0.002*). In total, 77.4% (48) of current smokers, showed strong or moderate LRG1 expression, whereas non-smokers had significantly lower expression levels. LRG1 expression was compared between smokers and non-smokers among patients with an HPV-positive OPSCC. There was no clear association between LRG1 and smoking.

Strong immunopositivity of LRG1 was observed in patients who reported to consume alcohol, compared to non-drinkers (*p* = *0.039*). In addition, LRG1 expression was stronger in tumors without lymph node metastasis compared with N + tumors (*p* = *0.025*). Stage III-IV tumors had stronger LRG1 expression compared with stage I-II tumors (*p* = *0.033*). Furthermore, LRG1 expression seemed to be related to low pathological grade *(p* = *0.015)*, but the material was scarce in this group.

***SDR16C5*:** Immunohistochemical results from 154 samples were available for a total of 157 samples. Strong expression of SDR16C5 was detected more commonly in early-stage tumors (T1-T2) but this failed to reach statistical significance.

***PIP4K2C*:** Immunohistochemical results from 155 samples were available from a total of 157 samples. No statistically significant differences were observed in the expression of PIP4K2C and clinicopathological variables.

***MVD*:** Immunohistochemical results from 152 samples were available for a total of 157 samples. We observed significantly stronger MVD expression in tumors with higher T class (T3-T4) compared to tumors with lower T class (T1-T2) (*p* = *0.027*). MVD expression was stronger in tumors staged N0 compared with N + (*p* = *0.045*).

### Survival according to immunohistochemical markers and HPV status

**Figure 4:**
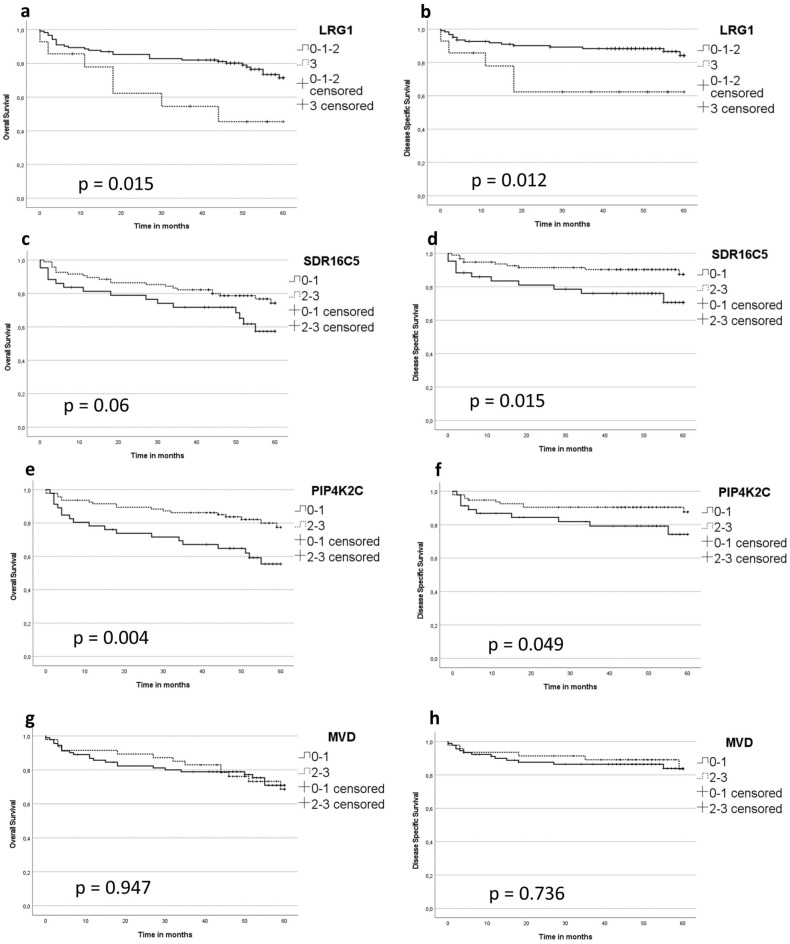
Overall survival (OS) (**A**, **C**, **E**) and (**G**), and disease specific survival (DSS) (**B**), (**D**), (**F**) and H according to protein expressions levels. LRG1 levels 0–1-2 as negative-weak-moderate, 3 as strong expression level. SDR16C5, PIP4K2C and MVD expression levels 0–1 as negative-weak and 2–3 as moderate-strong expression.

To define whether the expression levels of LRG1, SDR16C5, PIP4K2C and MVD are associated with patient survival, we evaluated both OS and DSS (Fig. 4). The median follow-up time was 60 months from the date of treatment completion to the date of last follow-up or death. We removed 15 patients with palliative treatment intent from the survival analysis.

***LRG1*:** Both OS (*p* = *0.015*) and DSS (*p* = *0.012*) were significantly more favourable among patients with lower LRG1 expression.

***SDR16C5*:** Patients with higher SDR16C5 scores had better DSS (*p* = *0.015*).

***PIP4K2C*:** Higher expression of PIP4K2C had a significant positive impact on both OS (*p* = *0.004*) and DSS (*p* = *0.049*).

***MVD*:** MVD expression level did not have a significant effect on OS or DSS.

Regression analysis by Cox method revealed that high PIP4K2C expression level (*p* =  < *0.001*) was independent prognostic factor in OS (Table 4).

**Table 4:** Risk factor analysis for overall survival by multivariate Cox regression.

Risk factor	*p* value	HR	95% Cl
PIP4K2C expression 2–3 vs. 1–0	< *0.001*	0.4	0.1–0.6
HPV status (neg vs. pos)	0.375	0.6	0.3–1.6
Stage III-IV vs. I-II	0.34	2.2	1.1–4.4
Age	*0.001*	1.1	1.0–1.1
Ex-smoker vs. never	0.885	1.1	0.3–3.7
Current smoker vs. never	*0.013*	3.9	1.3–11.5

*HPV* human papilloma Virus, *CI* confidence interval, *HR* hazard ratio.

## Discussion

In this study, our interest was to evaluate the expression and clinical impact of a group of novel proteins in HPV-positive and HPV-negative OPSCC tumors. We were interested to find out whether they might predict the histopathological behaviour of the tumors or patient survival. These proteins are known to be expressed in OPSCC patients’ serum but have never been specifically studied on OPSCC tissue samples.

LRG1 is a multifunctional pathogenic signalling molecule that interacts with the TGF pathway in a context-specific manner, among other functions^13,36^. In head and neck squamous cell carcinoma, LRG1 has been reported to be downregulated^21^. Interestingly, earlier studies have shown either upregulation or downregulation of LRG1 in oesophageal squamous cell carcinoma (ESCC)^37,38^. Our data show that the HPV status of the tumor correlates with the expression level of LRG1 in OPSCC. Using immunohistochemistry, we demonstrated stronger expression of LRG1 in HPV-negative than HPV-positive tumors. In addition, strong LRG1 expression was linked with smoking and current alcohol use but not as an independent factor. This is in line with previous studies that indicate smoking and alcohol consumption to be the two main risk factors for OPSCC and pathogenesis for HPV-negative tumors^39,40^. In our study, low expression of LRG1 was associated with better overall and disease-specific survival, phenomena previously linked to HPV-positive tumors in general^41–43^. Since LRG1 is a secreted protein, it would be interesting to study it in more detail in OPSCC patient sera and to examine more closely its expression in HPV-negative tumors.

According to our results in SCC cell culture samples SDR16C5 expression was stronger in HPV-negative cell lines. Contradictory, in immunohistochemical analyses of OPSCC tissue samples, SDR16C5 expression was stronger in HPV-positive tumors and associated with better disease-specific survival. This discrepancy warrants further studies to explain the phenomena behind it, although it is a known fact that tumor cell lines do not represent native tumor samples as such^44,45^. According to previous studies, higher SDR16C5 expression has been shown in pancreatic cancer as well^46^, but instead decreased expression in triple-negative breast cancer^47^ and in head and neck carcinoma^48^. In addition, SDR16C5 expression has been shown to be a risk factor for the prognosis of colorectal carcinoma ^49^. Thus, SDR16C5 appears to be differentially expressed in various cancers and its specific role is not yet clear.

Previous studies have found mutation of *TP53* and amplification of PIP4K2C co-occurrence in breast cancer^50^. In addition, mutation of *TP53* is often found in HPV-negative OPSCC tumors^51^. Loss of PIP4K2A and PIP4K2B has been shown to reduce tumor growth in p53^-^/^-^ mice^50^. We observed significant PIP4K2C protein overexpression in HPV-negative cell lines, which could suggest amplification of the PIP4K2C gene.

Additionally, loss of PIP4K2C has been identified as a contributory factor of liver metastasis, but it does not support metastasis in other organs outside the liver^52^. Furthermore, we demonstrated strong PIP4K2C expression as an independent factor for overall survival. Low PIP4K2C expression level has been shown to be a favorable prognostic factor for example in acute myeloid leukemia (AML) (ref. 25). The contribution of PIP4K2C to survival needs to be studied further because its´ presence may play a significant role in various malignancies. Our finding of PIP4K2C as an HPV-independent marker could add value to the research of OPSCC biology.

Mutations of the MVD gene have been reported to be involved in porokeratosis, a disorder of defective keratinization^27^. Previous studies have proposed that deficiency of isoprenoids might predispose to idiopathic inflammation of the skin^27^. It is well known that the keratotic lesions can proceed to cutaneous neoplasms, in most cases squamous cell carcinomas^53,54^. We showed that MVD expression was down-regulated in both SCC cell lines and most significantly in the HPV-negative cell line. This abnormal expression of the MVD protein may lead to disturbances in cell keratinization^27^ and thus possibly to the progression of SCC. It should also be noted that HPV-negative tumors are typically well keratinized^55^.

There are a few limitations in our study. We obtained discordant results between Western blotting and immunohistochemistry for some of the protein expressions. When estimating these results, it should be noted that the samples are handled differently in different applications, which may affect the exposure of epitopes on target proteins, possible altering the ability of the antibody to bind specifically to its target^44,45^. Additionally, protein expression analyzed in vitro in cancer cell lines lack interaction with other cells and the tumor environment.

## Conclusions

In this study, we investigated new proteins that could be related to the HPV status of OPSCC patient samples. We observed strong LRG1 immunoexpression in HPV-negative tumors and strong SDR16C5 immunoexpression in HPV-positive tumors. We observed correlation between low expression of LRG1, strong expression of SDR16C5 and PIP4K2C and patient survival. Strong PIP4K2C immunoexpression had independent significance for overall survival and may thus be an important prognostic factor and unrelated to HPV status.

### Supplementary Information

Supplementary Information.

## Data Availability

All data generated or analysed during this study are included in this published article (and its Supplementary Information files).
