# Supplementary material for: LRG1 and SDR16C5 protein expressions differ according to HPV status in oropharyngeal squamous cell carcinoma
Source: Sci Rep. 2024 Jun 19;14:14148. doi: 10.1038/s41598-024-64823-w (PMC11187215; doi:10.1038/s41598-024-64823-w)
Supplement: Supplementary file 1 — Supplementary Information. [file 41598_2024_64823_MOESM1_ESM.pdf]

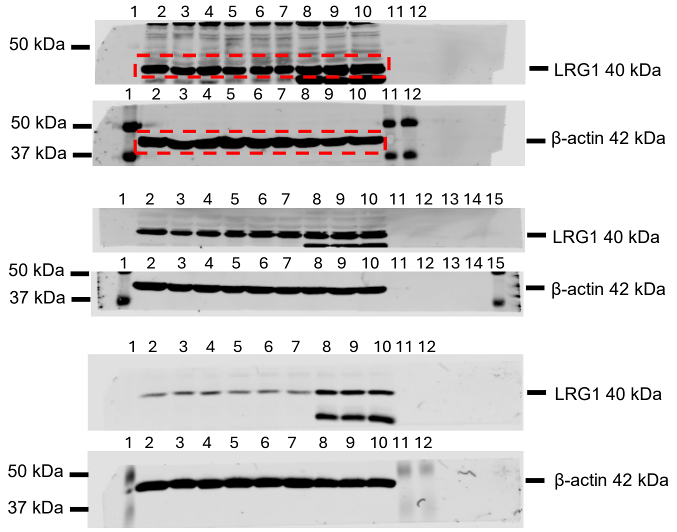
1 2 3 4 5 6 7 8 9 10 11 12
50 kDa
LRG1 40 kDa
50 kDa
37 kDa
β-actin 42 kDa
1 2 3 4 5 6 7 8 9 10 11 12 13 14 15
LRG1 40 kDa
50 kDa
37 kDa
β-actin 42 kDa
1 2 3 4 5 6 7 8 9 10 11 12
LRG1 40 kDa
50 kDa
37 kDa
β-actin 42 kDa

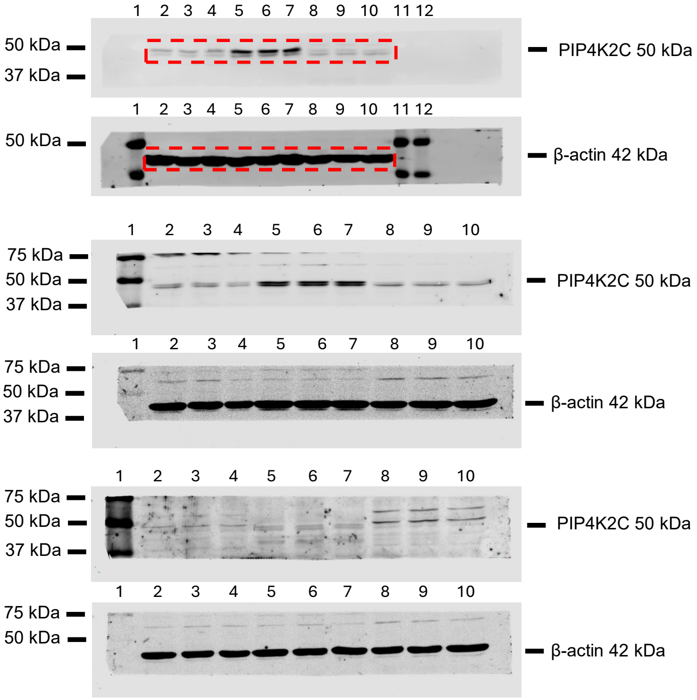
1 2 3 4 5 6 7 8 9 10 11 12
50 kDa
37 kDa
PIP4K2C 50 kDa
50 kDa
β-actin 42 kDa
1 2 3 4 5 6 7 8 9 10
75 kDa
50 kDa
37 kDa
PIP4K2C 50 kDa
75 kDa
50 kDa
37 kDa
β-actin 42 kDa
1 2 3 4 5 6 7 8 9 10
75 kDa
50 kDa
37 kDa
PIP4K2C 50 kDa
75 kDa
50 kDa
β-actin 42 kDa

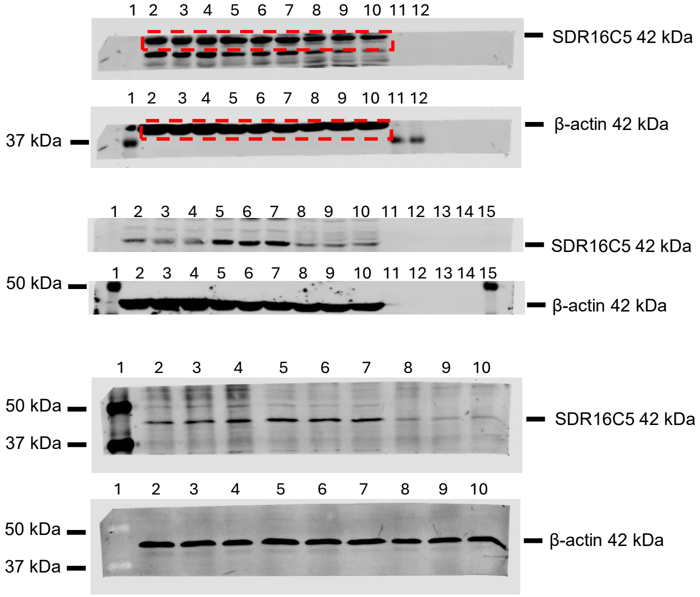
1 2 3 4 5 6 7 8 9 10 11 12
SDR16C5 42 kDa
37 kDa
β-actin 42 kDa
1 2 3 4 5 6 7 8 9 10 11 12 13 14 15
SDR16C5 42 kDa
50 kDa
β-actin 42 kDa
1 2 3 4 5 6 7 8 9 10
50 kDa
37 kDa
SDR16C5 42 kDa
50 kDa
37 kDa
β-actin 42 kDa

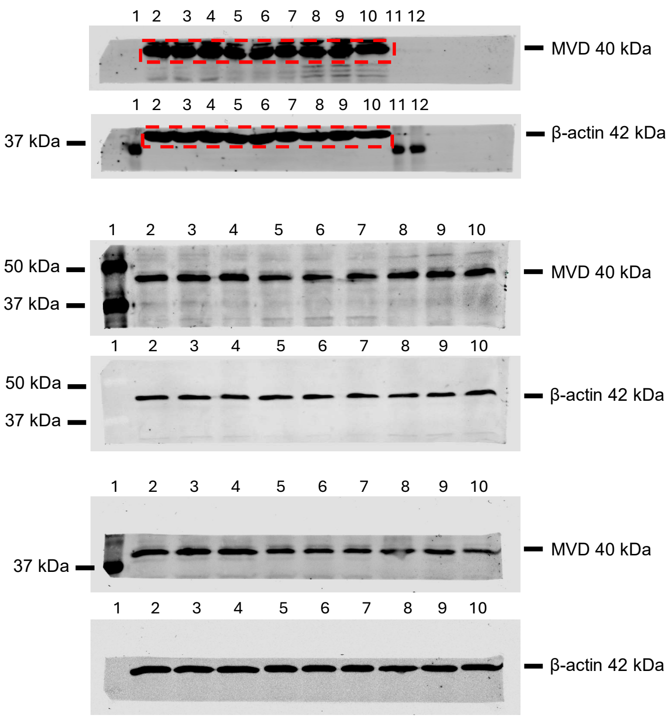

The order of the samples on the blots:

1. Molecular weight marker
2. HaCat
3. HaCat
4. HaCat
5. SCC-65
6. SCC-65
7. SCC-65
8. SCC-69
9. SCC-69
10. SCC-69
11. Molecular weight marker
12. Molecular weight marker
15. Molecular weight marker

} Only on some of the blots

- - - - - - - - - Red dashed line showing the cropped area for **Fig.2A**

Supplementary Fig. 1. Original Western blotting membranes of all experiments used for analyses. After incubation with the primary antibodies, the membranes were incubated with IRDye 800CW Donkey anti-rabbit/anti-mouse IgGs, IRDye 680RD anti-mouse IgG (LI-COR, Lincoln, NE, USA) or Alexa Fluor 680 Donkey anti-rabbit IgG (Invitrogen, Carlsbad, CE, USA) and scanned with the Odyssey Imaging System (LI-COR). The blots were scanned at different intensities for optimal quantification. Here, high-intensity grayscale images are shown to visualize the edges of the membranes and the molecular weight markers visible at the 680 nm channel. In order to probe for other proteins from the same membrane, the membranes were cut into smaller strips before incubating with the primary antibodies.
